# Implementation of carceral medicaid suspension and enrollment programs: perspectives of carceral and medicaid leaders

**DOI:** 10.1186/s40352-024-00311-7

**Published:** 2025-01-09

**Authors:** Sachini Bandara, Brendan Saloner, Hannah Maniates, Minna Song, Noa Krawczyk

**Affiliations:** 1 Department of Mental Health, Johns Hopkins Bloomberg School of Public Health, 624 N. Broadway Ave, Baltimore, MD 21205 USA; 2 Department of Health Policy and Management, Johns Hopkins Bloomberg School of Public Health, 624 N. Broadway Ave, Baltimore, MD 21205 USA; 3 National Association of Medicaid Directors, 601 New Jersey Avenue NW, Washington, DC 20001 USA; 4 Center for Opioid Epidemiology and Policy, Department of Population Health, NYU Grossman School of Medicine, 190 Madison Ave, New York, NY 10016 USA

**Keywords:** Medicaid, Criminal justice, Health policy

## Abstract

**Background:**

Medicaid expansion via the Affordable Care Act, more recent legislation and Medicaid 1115 waivers offer opportunity to increase health care access among individuals involved in the carceral system. Effective enrollment of new beneficiaries and temporary suspension and reactivation of existing Medicaid benefits upon release is key to the success of these efforts. This study aims to characterize how jails, prisons and Medicaid agencies are implementing Medicaid suspension and enrollment programs and identifies barriers and facilitators to implementation.

**Methods:**

We conducted 19 semi-structured interviews with 36 multi-state leaders in carceral facilities, Medicaid agencies, local health departments and national policy experts from 2020 to 2021. Interviews covered 4 domains: (1) the role of policy in influencing carceral and reentry Medicaid practices, (2) implementation strategies to suspend and enroll incarcerated individuals into Medicaid, (3) barriers and facilitators to successful implementation, and (4) variation in implementation between jails and prisons.

**Results:**

Participants identified logistical challenges with suspension and enrollment, including limited infrastructure for data sharing between carceral facilities and Medicaid agencies, burdensome bureaucratic requirements, and challenges with Medicaid renewal, particularly in the jail environment. They offered opportunities to overcome barriers, such as the creation of specialized incarcerated Medicaid benefit categories and provision of in-reach services via managed care organizations. Participants also called for improvements to Medicaid reactivation processes, as even when facilities successfully suspended benefits, individuals faced significant challenges and delays reactivating benefits upon release. Participants also called for further loosening of the Medicaid Inmate Exclusion Policy.

**Discussion:**

Findings highlight the need to update data sharing infrastructure, which will be critical to the implementation of the 1115 waivers, as carceral facilities will be subject to Medicaid billing and reporting requirements. In addition to investing in the ability to newly enroll and suspend Medicaid benefits, attention towards improving timely reactivation practices is needed, particularly given the highly elevated risk of mortality immediately after release. Participants calls for further reforms to the Medicaid Inmate Exclusion Policy are consistent with proposed legislation.

**Conclusions:**

Findings can critically inform the successful implementation of Medicaid-based reforms to improve the health of incarcerated and formerly incarcerated people.

## Introduction

In 2021, over 1.2 million individuals were incarcerated and 440,000 were released from federal or state prison (Carson, 2022). In the same year, 619,000 individuals were incarcerated on an average day in local jails with 6.9 million annual admissions annually (Zeng, 2022). Incarceration may present an opportunity to initiate health care for some but is also associated with service disconnection and deterioration of physical and mental health outcomes (Wildeman & Wang, 2017). These declines persist after release with formerly incarcerated individuals experiencing higher rates of physical, mental and substance use related illness and poorer access to health care and social services compared to the general population (Wildeman & Wang, 2017). The period immediately following incarceration is a highly vulnerable time. The risk of death in the year following release, specifically overdose death, is extremely elevated (Binswanger, 2013; Merrall et al., 2010).

One policy mechanism for mitigating these negative health risks during the reentry period has been to expand access to insurance coverage following release, (Barnert et al., 2022) specifically through Medicaid. Under the Affordable Care Act, most individuals being released from jail and prison in Medicaid expansion states are eligible for Medicaid due to their low incomes (Guyer et al., 2019). Prior research has documented early jail and prison efforts to enroll individuals into Medicaid as part of the reentry process, (Bandara et al., 2015) and more recent literature has found that having coverage following release is associated with increased use of health care services, reduced recidivism and faster access to care (Badaracco et al., 2021; Balio et al., 2021; Burns et al., 2022; Saloner et al., 2022).

Medicaid’s reach has been limited to the post-release period, because of the Medicaid Inmate Exclusion Policy, which prohibits the use of Medicaid funds to pay for health care services received while incarcerated, except for offsite inpatient services (The Social Security Act Ammendments, 1965). Because of this policy, Medicaid benefits were historically terminated upon incarceration, often leaving individuals without coverage upon release. More recently in an effort to further improve post-incarceration coverage, states have been enacting policies to temporarily suspend (rather than terminate) benefits and reactivate benefits upon release (Bryant, 2019). As of 2019, over 40 states had policies to temporarily suspend Medicaid benefits for individuals incarcerated in jails or prisons (Kaiser Family Foundation, n.d.). However, implementation of these policies is a vast undertaking and relies on effective communication and data sharing between disparate carceral authorities and Medicaid agencies and likely varies widely. There has been limited research on the implementation of these suspension policies at the jail and prison level, and as the Affordable Care Act’s Medicaid expansion approaches 10 years post-enactment, shifts in implementation practices of carceral enrollment programs for new beneficiaries are still not well understood.

Recent policy changes have granted states new flexibilities around the Medicaid Inmate Exclusion Policy, allowing coverage of individuals who are incarcerated through Sect. 1115 demonstration projects, which allow the Secretary of Health and Human Services to waive typical restrictions on Medicaid programs. Recent guidance from the Center for Medicare and Medicaid Services (CMS) outlines how the 1115 waiver process can be used to provide Medicaid-funded health care services in the 90 days immediately prior to release to improve care transitions for high-need beneficiaries (Tsai, 2023). This represents the first partial waiver of the Medicaid Inmate Exclusion Policy (Haldar & Guth, 2023). As of August 2024, 11 states have such waivers approved and 13 other states have similar waivers pending (Kaiser Family Foundation, 2024). In addition, the Omnibus Consolidated Appropriations Act of 2023 require state Medicaid agencies cover case management and health screenings for incarcerated individuals 21 years and younger in the 30 days prior to release (Health and Reentry Project, 2023). Guidance on implementation of this law is forthcoming. Key to the success of these reforms to provide Medicaid-covered services during incarceration will be the ability of jails and prisons to enroll new beneficiaries into Medicaid and effectively suspend and reactivate existing benefits.

This study aimed to inform ongoing efforts to improve Medicaid coverage of individuals involved in the carceral system by characterizing implementation of Medicaid suspension and enrollment within jails and prisons. Through key informant interviews with multi-state leaders of jails, prisons, Medicaid agencies and national policy experts, this study elucidates barriers to implementing Medicaid suspension and enrollment processes in jails and prisons and identifies important considerations for improving the effectiveness and success of these programs.

## Methods

We recruited 4 types of participants with expertise on Medicaid suspension and enrollment practices, including: (1) leaders in carceral systems responsible for overseeing Medicaid suspension and enrollment, including medical directors, reentry coordinators and data analysts; (2) leaders at state and county Medicaid offices responsible for working with carceral systems; (3) local and state health department officials responsible for facilitating relationships between Medicaid agencies and carceral facilities; and (4) national experts on Medicaid suspension and enrollment, including federal government officials, academic researchers, and technical assistance providers. Leaders from carceral systems, Medicaid offices and health departments were recruited from 5 states, 1 that was consistently identified by national experts, grey and academic literature as having high performing carceral Medicaid suspension and enrollment programs and 4 states participating in the Bloomberg Overdose Prevention Initiative, a multistate campaign to support overdose prevention efforts funded by Bloomberg Philanthropies. All states had a carceral system with separate county and state-run facilities, rather than a unified state-run system. The average number of incarcerated people in jails and prisons per 100,000 residents in each state ranged from 310 to 860. (Minton et al., 2021) Snowball sampling was also used to identify respondents within states with expertise in carceral suspension and enrollment programs. Interviews were conducted via videoconferencing and lasted approximately 45 min. Interviews were audio recorded and transcribed for analysis.

We conducted semi-structured interviews covering 4 domains: (1) the role of policy in influencing carceral and reentry Medicaid practices, (2) implementation strategies to suspend and enroll incarcerated individuals into Medicaid, (3) barriers and facilitators to successful implementation of Medicaid suspension and enrollment, and (4) variation in implementation between jails and prisons. Transcripts were coded using a hybrid inductive-deductive coding approach. Two study team members piloted the codebook by double-coding 5 transcripts and refined the codebook through organizing and developing themes. The remaining transcripts were coded by a single study team member using the final codebook. Data collection and analysis were guided by the Consolidated Framework for Implementation Research (CFIR), a framework to evaluate policy and program implementation (Damschroder et al., 2009). Domains of CFIR informed the development of the interview guide and an initial set of codes during the double coding phase. Interviews were conducted from September 2020 to May 2021. Research protocols were approved by the Johns Hopkins Bloomberg School of Public Health Institutional Review Board.

## Results

### Sample characteristics

We conducted 19 interviews with 36 participants, with several interviews including multiple individuals at a time. At the organization level, interviews represented 8 jails, 5 Medicaid agencies, 3 local health departments and 3 national expert organizations. Participants included 12 carceral officials, 14 Medicaid officials, 5 national experts, and 5 local health department officials. Geographic distribution varied, with 16 participants in the Middle Atlantic Census division, 12 in the South Atlantic division, 7 in the East North Central division, and 1 participant in the Mountain division. All respondents resided in states with Medicaid expansion and suspension policies as determined by legal mapping from Kaiser Family Foundation (Kaiser Family Foundation, n.d.). The themes outlined below were consistently reported across type of respondent (carceral, Medicaid, health, national expert).

### Role of federal policy

Federal policy was frequently cited as influencing efforts to suspend and enroll individuals into Medicaid (themes and relevant quotes displayed in Table 1). Most respondents discussed how complying with the Medicaid Inmate Exclusion Policy resulted in states and localities having to suspend or terminate Medicaid upon incarceration. Several participants discussed a desire for increased flexibilities to the Medicaid Inmate Exclusion Policy, particularly around the timing of when Medicaid benefits needed to be suspended upon incarceration. Participants described the inefficiency of suspending and reactivating individuals who are incarcerated in jails multiple times a year for a short period. They also described frustration for individuals getting coverage suspended over a very short incarceration and then having to spend much longer reactivating coverage. Several carceral officials discussed purposively delaying reporting of incarcerations to Medicaid agencies to prevent these scenarios and reduce reporting burden for the carceral facilities. However, participants were reluctant to make these delays in reporting a formal practice, out of fear of violation of federal Medicaid policy.

**Table 1:** Role of Federal Policy in Medicaid suspension and enrollment

Themes	Representative Quote
Inmate exclusion creates need for enrollment and suspension activities	“We’ve talked about a number of things that are quite technical that may be seeming around on the edges, but it is working around this statutory requirement, which is the payment exclusion. And so our focus becomes, how quickly can somebody get back in? How quickly can you suspend in order to comply?” —National Expert
Desire for more flexibility on Medicaid Inmate Exclusion Policy	“At the local level there are people who they see three to five times a month… Additional federal flexibilities would be really helpful there” —Medicaid Official
New 1115 Medicaid waivers will spur more better suspension and enrollment programs	**“**I think that if you’re a state and you’re trying to decide whether to make the investments that are needed to get people who are incarcerated, enrolled in Medicaid, the fundamental question you ask yourself is ‘what is the cost benefit analysis?’ And if you’re able to provide services to people and enhance the connections that they face at entry or reentry between health care services, I think the business case becomes much stronger and the benefits become much stronger” —National Expert
Calls to consider exemption of pre-trial detainees from Medicaid Inmate Exclusion Policy	“If somebody is still in pretrial why would you shut it off? Frankly, I think it’s an easier solution … jails barely have good data to know who’s coming in and out… it is just easier for them to just know this person you’re still pre-trial has not been charged or convicted” —National Expert

Several participants cited the potential of Medicaid 1115 waivers to improve suspension and enrollment policies. In particular, the ability of the waivers to allow access to new federal and state funds for carceral health care services was cited as motivation for carceral facilities and Medicaid agencies to improve suspension and enrollment processes and policies. A few participants supported further loosening of the Medicaid Inmate Exclusion Policy to allow for the use of Medicaid funds who are incarcerated pre-trial and not yet convicted of a crime.

### Medicaid suspension and enrollment implementation strategies

Participants described several models of Medicaid enrollment and suspension programs when these activities occurred in facilities. Larger jail and prison facilities reported integrating Medicaid activities into broader intake and release planning activities and staffing by personnel responsible for those functions and having automated data sharing capabilities with Medicaid offices. Smaller facilities reported having a single staff member responsible for identifying incarcerated individuals’ Medicaid eligibility status, assisting in enrollment and reporting suspension to Medicaid officials.

Participants outlined several strategies to implement effective Medicaid suspension and enrollment in carceral facilities (Table 2). Many participants described the state’s creation of a specialized Medicaid benefit category for incarcerated individuals that did not allow for the use of Medicaid funds for health care services except for inpatient hospitalizations. This category facilitated both suspension and new enrollment, as existing Medicaid beneficiaries could be moved to this benefit category upon incarceration and new beneficiaries could be newly enrolled into this benefit category prior to release.

**Table 2:** Medicaid suspension and enrollment implementation strategies

Theme	Representative Quote
Wide varieties of strategies to implement suspension and enrollment	“[Suspension] is not really a concept in the regulation. It is a way to effectuate the payment exclusion. And so, from an operational standpoint, a state [could] come up today with a completely different way that achieved the same purpose.” —National Expert
Creation of specialized inmate Medicaid benefit category	“Anyone who is active when they come in will get switched to this incarceration benefit plan which limits the type of services that we’ll pay for to offsite inpatient stays.” —Medicaid Official
Benefits of pre-release enrollment	“If we get the paperwork done as soon as the person enrolls in the program, or a minimum of 30 days before they walk out of the door, at any point we can just hand that paper off to a Medicaid worker who can turn the benefits on.” —Carceral Official
Simplifying application process facilitates new enrollments	“I think the old application was like 20 pages, and this one was less than a page. So that was a huge contribution.” —Carceral Official
In-reach programs prior to release	“It’s just a much stronger approach, particularly for someone who’s living in incarceration, which is an unbelievably vulnerable time in a person’s life and they have a lot going on. Actually giving them the connection to care and services is much, much more likely to move the needle than an eligibility-alone approach is.” —National Expert

Some respondents reported challenges to submitting new Medicaid enrollment applications prior to an individual’s release, reporting that applications were denied by Medicaid offices due to incarceration status. One strategy to overcome this was having state Medicaid agencies explicitly allow for enrollment prior to release was allowed by state Medicaid agencies, which was consistently reported as a key for successful implementation of enrollment programs. Implementation strategies for pre-release enrollment included the ability to enroll individuals into specialized Medicaid benefit categories, collaboration between Medicaid and carceral facilities to create technological infrastructure that allowed for pre-release enrollment and collecting all necessary documentation and consent for enrollment as part of the carceral intake process. Participants also reported the benefits of simplifying the Medicaid application process to reduce burden for carceral facilities and improve implementation. This included significantly shortening the Medicaid application form, allowing carceral systems access to web-based application systems, and changing consent forms during intake to opt out instead of opt-in.

Another innovation highlighted by participants was building in contractual requirements for managed care organizations (MCOs) to provide in-reach care coordination services for members who are incarcerated 30 days prior to release. Under current Medicaid regulations, managed care organizations have flexibility to provide these types of services as part of the capitation rate they receive from states. In some states this was required only for beneficiaries deemed to have high needs, like chronic health conditions. Respondents consistently highlighted the value of these services in helping to improve linkage to care post release and maintain enrollment in Medicaid post-release. These services were often limited to individuals who had a verified release date and sometimes limited to individuals enrolled in an MCO at the time of incarceration. Other innovations included legislative action to require Medicaid enrollment and suspension programs for carceral facilities and enactment of presumptive eligibility for incarcerated individuals, which allowed for carceral facilities to gather basic income and disability information from an individual and immediately enroll individuals into temporary Medicaid coverage without having to wait for full determination from a Medicaid agency.

### Barriers and facilitators to implementation success

Participants highlighted several barriers and facilitators to the successful implementation of Medicaid suspension and enrollment programs in carceral facilities (Table 3). Effective data sharing capabilities between carceral facilities and Medicaid offices was universally highlighted as critical to the successful implementation of Medicaid suspension and enrollment programs. Outdated data sharing systems often created cumbersome obstacles: while some state prisons had automated data transfer to inform Medicaid agencies of which beneficiaries to suspend or reactivate due to changes in incarceration status, other carceral facilities employed manual systems of data sharing via in person deliveries or email. Even when a state level suspension policy was theoretically in place, in some cases benefits were still mistakenly terminated or left active due to lack of data sharing capabilities. Lack of funding to develop technological infrastructure and hire staff were highlighted as barriers to success. Engaged leadership within state government, carceral facilities and Medicaid agencies that could address such issues were routinely cited as key facilitators for success.

**Table 3:** Barriers and facilitators to Medicaid Suspension and enrollment implementation

Theme	Representative Quote
Data sharing between Medicaid agencies and carceral facilities is critically important	“The real turning point for it taking off was when we gave [carceral facilities] access to the online [enrollment] portal. When we started automating those processes, then a lot more counties were on board because processes were happening behind the scenes. Prior to that, there were a lot of manual processes that were going on like paper applications being submitted and people are having to review that. And with our online portal, many of those steps are taken care of automatically.” —Medicaid Official
Lack of funding and resources inhibited success	Once you’re inside a jail like our jail, they have very limited resources. It’s extremely chaotic. They’re very busy.” —Carceral Official
Engaged leadership facilitated success	“We have the support of the governor’s office. We have support of the director in [redacted] Department of Corrections…it’s a policy gift.” –Medicaid Official
Challenges with reactivation of benefits following suspension	“The benefits [are] being suspended but not reactivated… in my experience, it is never turned back on automatically in the manner that it’s supposed to. [Redacted name of jail reentry coordinator] has to physically take his referrals to the Board of Social Services to have the benefits reinstated.” –Carceral Official
Medicaid renewal during incarceration often overlooked	“There’s a tendency to focus on application. But there’s also a renewal process. So if you come to prison or jail and you already have Medicaid coverage, or if you’re in prison for a long time, your coverage gets renewed every 12 months. But that very rarely happens in a corrections context.” –National Expert
Suspension and enrollment helped with other reentry programs	In regards to [medications for opioid use disorder] and linkage of the community, in starting all this, we found that the community partners were not willing to make partnerships unless we had some level of [Medicaid] enrollment process for them so that by the time the patient showed up, either an application was in place or they had coverage already.” –Carceral Official

In some jurisdictions where benefits were successfully suspended, participants reported that incarcerated individuals could still face challenges with reactivation upon release. While some participants reported immediate automatic reactivation of Medicaid benefits upon release, participants in other states reported that formerly incarcerated individuals were required to physically go to Medicaid offices to reactivate benefits upon release or wait several weeks for reactivation to occur due to processing delays by Medicaid.

Renewal of benefits during incarceration for beneficiaries whose Medicaid was suspended was frequently cited as being overlooked. For example, if an individual is incarcerated for over a year, their Medicaid eligibility would generally need to be renewed. If the state Medicaid agency cannot automatically renew the individual’s eligibility using available data sources (often called an ex parte renewal), the state would send the individual a paper renewal form. However, if the state and carceral facility do not have a process to ensure the incarcerated individual receives and completes this renewal form, the individuals’ suspended Medicaid would effectively be terminated.

Another facilitator for expansion of more effective carceral Medicaid suspension and enrollment programs were the benefits these programs had on other reentry and healthcare initiatives. For example, some participants cited that efforts to ensure Medicaid access upon release positively affected programs that provide medications for opioid use disorder. These medication programs often set up appointments with community-based providers for patients with an imminent release, and finding such appointments were reportedly easier when carceral facilities could confirm that patients would have active Medicaid benefits upon release.

### Variation between jails and prisons

Participants highlighted several implementation considerations that were unique to county jails versus state-run unified or prison systems (Table 4). Almost all participants confirmed that Medicaid suspension and enrollment was more challenging in county jail environments due to several factors. The first was high turnover in jails, with many individuals incarcerated in jails for short periods of times, sometimes less than 48 h, and released at unanticipated times. The high volume of individuals whose Medicaid benefits must be suspended and activated quickly proved to be very burdensome for jail facilities and unanticipated release dates did not allow jail staff to plan ahead for Medicaid activation. This contributed to jails being less likely to prioritize Medicaid suspension and enrollment programs and more likely to not report incarceration to Medicaid offices thereby not initiating suspension. High turnover also resulted in less focus on addressing medical needs that were not acute, and therefore participants perceived there was less priority placed on programs that could facilitate long term health care access, such as Medicaid enrollment programs.

**Table 4:** Considerations for implementation of Medicaid suspension and enrollment in Jails

Theme	Representative Quote
Short stays and unpredictable release dates create challenges	“The biggest operational challenge with jails is just that people are in and out all the time. And the length of stay– the average stay is 25 days, but half of the population turns over every week.” –National Expert
Differences in priority	“[Medicaid enrollment is] basically the last thing they’re really thinking about. So, they’re not really addressing their medical because they’re anticipating a short term stay. So it’s not really a first priority.” –Medicaid Official
Statewide coordination difficult across jails	“The ability to partner and coordinate, and at times, have things really driven by the governor’s office, makes prisons an easier lift because you’re accountable to the same entity.” –National Expert
Jails need relationship with local Medicaid office	“We actually have a pretty good working relationship with our county assistance office so we can submit [Medicaid] applications up to 30 days before an individual is released. And most if not– I would say the majority of cases, the inmate will be activated the day the individual is released. So we’re really fortunate in that respect.” –Carceral Official
Need to rely on low-tech solutions	“On the jail side is all manual. We have to be told when they go in, and we have to be told when they leave. And if anything breaks down in terms of the communication on the back end, then we’ve got incorrect information.” –Medicaid Official
Rural jails face particular challenges	“In some of the more rural counties, they just don’t have the population to do the enrollment suspension and to do the pre-release inmate applications. It requires the data agreements. It requires manpower. And so some of the more rural counties just don’t have the population to– at least from the county sheriff’s perspective, they may not appreciate the benefits to justify the expenditures.” –Medicaid Official

Many participants perceived that state prisons and unified systems being part of the same level and branch of government as state Medicaid agencies facilitated their ability to create successful Medicaid suspension and enrollment programs. Participants highlighted the benefits of both entities having more resources than local agencies that oversee jails, perceptions that data sharing across 2 state-level agencies was easier, and the logistical ease of coordinating between 2 entities rather than several local entities. They also discussed that both state run carceral systems and Medicaid agencies reported to Governors’ offices, which allowed for easier coordination and the ability for the Governors’ offices to compel action.

Participants, particularly jail officials, reported that strong relationships with local branches of the state Medicaid office were key to the success of their Medicaid suspension and enrollment programs. While state-run carceral facilities were more likely to have automated systems in place for suspension and enrollment, several participants described homegrown systems at the local level. For example, several jails described relying on emailing lists of incarcerated individuals directly to specific county Medicaid officials with whom they had long standing relationships who would then suspend or activate Medicaid benefits when needed. This system also exemplifies another common theme highlighted by participants, in which jails often relied on low-tech solutions to implement their Medicaid suspension and enrollment programs. While this helped mitigate barriers to entry given the lack of data infrastructure at the jail level, it was also more subject to human error. A few participants who worked at county run carceral or Medicaid offices called for more state guidance and standards for jails on how to implement successful Medicaid suspension and enrollment programs. In addition to issuing such guidance, 1 state also created a centralized state-level Medicaid eligibility determination team for local jails to avoid variation in practices by county.

National experts and state Medicaid officials, who had experience working with both rural and urban jails, also discussed how rural jails faced unique challenges to implementing Medicaid suspension and enrollment programs. Because incarcerated populations and staffing levels were smaller, but programs still required establishing data sharing relationships with Medicaid agencies, many rural jails did not conduct any Medicaid suspension or enrollment activities despite existing state Medicaid suspension policies.

## Discussion

This study highlights several aspects of the implementation of carceral Medicaid suspension and enrollment programs that should be considered in the midst of recent policies to expand access to health care services for incarcerated populations. Consistent with prior research on programs enrolling new Medicaid beneficiaries soon after enactment of the Affordable Care (Bandara et al., 2015; Bechelli et al., 2014; Grodensky et al., 2018; Riedel et al., 2016), we find limitations due to logistical constraints. Participants reported that unpredictable releases, a lack of resources, issues with technology, and bureaucratic requirements related to the Medicaid Inmate Exclusion policy inhibited implementation of both enrollment and suspension programs. However, study participants highlighted several strategies that may assist in overcoming these barriers, including the creation of specialized Medicaid benefit categories for incarcerated populations, allowing pre-release enrollment, and establishing strong relationships between Medicaid and carceral agencies.

In addition to improving the effectiveness and efficiency of current Medicaid suspension and enrollment programs, these strategies will be important to the successful implementation of new CMS 1115 waivers that allow for Medicaid-funded services prior to release. CMS guidance stipulates that states receiving these waivers must provide case management to assess and address medical and social needs, medications for opioid use disorder and a month’s supply of medication upon release and that services can be provided up to 90 days prior to release (Tsai, 2023). Specialized benefit categories and in-reach managed care services identified in this study can serve as foundations for the provision of these benefits. Our study highlights the need to update data sharing infrastructure, particularly in jails. Lack of data infrastructure has resulted in some carceral facilities relying on personal relationships with Medicaid officials to execute enrollment and suspension activities, rather than automated processes that are less subject to instability during worker turnover or limited ability to scale. An infrastructure update is also critical to the implementation of the 1115 waivers, as carceral facilities providing services under these waivers will be subject to Medicaid billing and reporting requirements, and will therefore a need to implement electronic health record and claiming systems to execute these requirements (Saloner, 2023). At minimum, states with approved 1115 waivers will be expected to report measures of the number of individuals screened for eligibility for 1115 services, utilization of pre-release and post-release services, the number of participants with care plans established at release and quality of care and health outcome metrics for participants (Tsai, 2023) It is therefore encouraging that in already approved waivers CMS has allowed the use of a portion of waiver funds to build data infrastructure and collaboration between agencies (Haldar & Guth, 2023).

This study also highlights how simply having a state-level Medicaid suspension policy does not guarantee that suspension and timely reactivation will occur. This is consistent with pre-ACA studies that find variation in suspension practices and the length of reactivation across states (Morrissey et al., 2006; Rosen et al., 2014). In addition to investing in the ability to newly enroll and suspend Medicaid benefits, attention towards improving timely reactivation practices is needed, particularly given the highly elevated risk of mortality and other hardship experienced among formerly incarcerated in the 2 weeks following release (Binswanger, 2013; Merrall et al., 2010).

Finally, participants of this study call for further loosening of the Medicaid Inmate Exclusion Policy beyond what was done by the 1115 waivers, such as expanding access to Medicaid for pre-trial detainees and loosening the timing of when Medicaid suspension must occur. This is consistent with recommendations from experts on carceral health (Barnert et al., 2022; Khatri & Winkelman, 2022) and would ease logistical burdens for carceral facilities and prevent disruptions in coverage, particularly for individuals who are incarcerated for short periods. Though political will to fully eliminate the Medicaid Exclusion Policy has been historically limited, 3 current bipartisan proposed pieces of federal legislation seek to loosen this policy. The Due Process of Continuity of Care Act would allow pretrial detainees to access Medicaid benefits while incarcerated. The Reentry Act would allow Medicaid funded services 30 days prior to release regardless of 1115 waiver status, and the Humane Correctional Health Care Act would eliminate the Medicaid Inmate Exclusion policy all together (Health and Reentry Project, 2023). While these legislations are not yet enacted, they speak to increased policy attention towards improving healthcare access of incarcerated individuals using Medicaid.

### Limitations

Several study limitations should be considered. Participants were national experts or from states with Medicaid suspension and expansion policies and may not be generalizable to a broader population of carceral and Medicaid officials. Second, interviews were conducted prior to the release of the 2023 cm guidance on Medicaid 1115 waivers to provide partial exemption to the Medicaid Inmate Exclusion Policy, which may have shifted implementation practices. Third, data may be subject to social desirability bias; however, participants were assured anonymity and interviews were designed to promote candid conversation to address this limitation. Fourth, interviews did not discuss the role of private prisons and private carceral health care organizations, which may be relevant to Medicaid enrollment and suspension programs.

## Conclusion

These policies under consideration and currently enacted reforms via the Affordable Care Act, Medicaid 1115 waivers and Omnibus Consolidated Appropriations Act of 2023 provide important steps in improving access to insurance for a highly vulnerable and marginalized population. However, our study highlights that achieving the promise of these reforms will require investment in infrastructure, technological capacity, staffing, clear guidance and other resources to ensure that implementation effectively fulfills policy goals.

## Data Availability

The datasets used and/or analyzed during the current study are available from the corresponding author on reasonable request.

## Abbreviations

CMS: Center for Medicare and Medicaid Services
CFIR: Consolidated Framework for Implementation Research
MCO: Managed care organizations
